# The Immunogenicity and Anti-tumor Efficacy of a Rationally Designed Neoantigen Vaccine for B16F10 Mouse Melanoma

**DOI:** 10.3389/fimmu.2019.02472

**Published:** 2019-11-05

**Authors:** Yan Zhang, Zhibing Lin, Yuhua Wan, Huaman Cai, Li Deng, Rongxiu Li

**Affiliations:** ^1^State Key Laboratory of Microbial Metabolism, School of Life Sciences and Biotechnology, Shanghai Jiao Tong University, Shanghai, China; ^2^Shanghai HyCharm Inc., Shanghai, China; ^3^Engineering Research Center of Cell and Therapeutic Antibody, Ministry of Education, Shanghai, China

**Keywords:** cancer vaccine, immune response, tumor neoantigen, B16F10 melanoma, helper T cell, cytotoxic T lymphocytes

## Abstract

Tumor neoantigens are ideal targets for cancer immunotherapy as they are recognized by host immune system as foreigners and can elicit tumor-specific immune responses. However, existing strategies utilizing RNA or long peptides for the neoantigen vaccines render limited immune responses since only 20–30% of neoantigens predicted *in silico* to bind MHC I molecules are capable of eliciting immune responses with the majority of responding T cells are CD4^+^. Therefore, it warrants further exploration to enhance neoantigen-specific CD8^+^ T cells responses. Since neoantigens are naturally weak antigens, we asked whether foreign T help epitopes could enhance their immunogenicity. In present study, we chose 4 weak B16F10 neoantigens as vaccine targets, and fused them to the transmembrane domain of diphtheria toxin, namely DTT-neoAg. Strikingly, the vaccine elicited anti-tumor CD8^+^ T cells responses and enhanced tumor infiltration of both T cells and NK cells. Impressively, DTT-neoAg vaccine significantly deterred tumor growth with the inhibition rate reached 88% in the preventive model and 100% in the therapeutic model at low dose of tumor challenge. Furthermore, after second challenge with higher dose of tumor cells, 33.3% of the immunized mice remained tumor-free for 6 months in the therapeutic model. Because DTT is a non-toxic domain of diphtheria toxin, it may be not of great concern in terms of safety as a Th epitope provider. Thus, the fusion strategy employed by this study may become a feasible and powerful approach for development of personalized cancer vaccines.

## Introduction

Genomic mutations altering signal transduction pathways that control cell proliferation or apoptosis can cause development of cancers (1). The mutated proteins can give rise to novel antigens, so called neoantigens when they are processed and presented to T cells (2). Neoantigen-specific T cells are found in tumors as well as in peripheral blood of cancer patients (3), and they are the principle mechanism that underlies clinical responses to many standard treatments and immunotherapeutic interventions including checkpoint blockade (4) and adoptive T cell transfer (5).

Tumor neoantigens are attractive targets for cancer vaccine design (6). Both preclinical (7–11) and early phase clinical studies (12–14) found that neoantigen-based poly-epitope vaccines can substantially expand the tumor-specific T cell pools, and steer the immune system to the selective destruction of cancers with limited off-target toxicities, which leads to cancer regression and long-term tumor-free survival.

With the advance of next generation sequencing technology, non-synonymous mutations can be identified by whole exome sequencing, and their expression can be determined by RNA-Seq (8). Nevertheless, to choose the right neoantigen epitopes for the vaccine design is not an easy task at all as over 90 percent of neoantigens are patient-specific (2) and multiple neoantigens are needed to address clonal heterogeneity of tumors (15). In addition, only a limited fraction of non-synonymous mutations can naturally lead to activation of neoantigen specific CD4^+^ (16, 17) or CD8^+^ (18–20) T cells. These cells are detectable within tumor-infiltrating lymphocytes or in peripheral blood.

Candidate mutation peptides with high affinity to MHC I molecules can be identified with high confidence by sensitive computation algorithms (21). However, only 20–30% of MHC I neo-antigen peptides can stimulate T cell responses by vaccination. Surprisingly, over 90% of the immunogenic MHC I peptides elicit CD4^+^ T cell responses (7, 8, 22). Although it has been confirmed that some of the neoantigen-specific CD4^+^ T cells are able to kill tumor cells, the majority of tumor-specific killer T cells identified in patients have been of CD8^+^ T cells origin (23).

In this study, we asked whether fusion of low-immunogenic neoantigens to DTT, a membrane translocation domain of diphtheria toxin, could enhance antigen-specific immune responses, in particular, CD8^+^ cytotoxic T cell responses. DTT has been shown to enhance immune responses to self-molecules (24, 25). Indeed, we found MHC I-binding mutation peptides of B16F10 melanoma that failed to elicit cytotoxic T cell responses become highly immunogenic when they are fused to DTT. In addition, we show that the fusion antigens can elicit tumor-specific CD8^+^ cytotoxic T lymphocytes when formulated with CpG and Alum adjuvants, and enhance CD8^+^ T cells and NK cells' infiltrating into tumor. This strategy would significantly expand the pool of candidate neoantigens, improve the effectiveness of the neoantigen vaccines and reduce the cost of patient-oriented vaccine design.

## Materials and Methods

### Mice, Mouse Melanoma Cell Line, Adjuvants

C57BL/6 mice (female, 6–8 weeks old, average weight 20 g) were purchased from Slaccas Laboratory Animal Inc. (Shanghai, China), and were housed in a climate controlled facility. All animal studies were performed in accordance with the guidelines approved by the Institutional Animal Care and Use Committee of Shanghai Jiao Tong University. Mouse melanoma cell line B16F10 was purchased from the Cell Bank of Chinese Academy of Sciences (Shanghai, China), and cultured in DMEM (Gibco, USA) supplemented with 10% FBS (Gibco, USA), 100 U/mL penicillin and 100 μg/mL streptomycin (Gibco, USA) at 37°C under a humidified atmosphere of 5% CO_2_. Aluminum hydroxide gel (Alum) was purchased from Invitrogen (Invitrogen, USA). CpG ODN1826 (TCCATGACGTTCCTGACGTT) was synthesized by HuaGene (Shanghai, China). All chemical reagents were of analytical grade.

### Neoantigen Selection

Four neoantigens were selected for this study based on a recent report from the laboratory of Sahin (7). These neoantigens are expressed in B16F10, and can bind to MHC I, but they are not immunogenic. For validation their existence in our working cell line, the mutated gene fragments were amplified from B16F10 genomic DNA, subjected to Sanger sequencing, and the cognate mutations were confirmed (data not shown).

### Expression Vector Construction

Four neoantigens, each contains 27 amino acids with the mutant positioned in the center. The neoantigens are fused in tandem via SG linkers. The DNA fragment encoding the neoantigen fusion was chemically synthesized and cloned into pUC57 (Huagene, Shanghai, China). DTT DNA fragment encoding the amino acid residues 202–378 of diphtheria toxin was previously described (25). The DTT fragment and the neoantigen fusion fragment were connected by GGGGSGGGGS linker sequence with DTT at N-terminal. The corresponding DNA fragment was generated by overlapping PCR with primers listed in Supplementary Table 1. DTT was amplified from pUC19-DTT with primers DTT-F and DTT-R. Neoantigen fusion gene was amplified from pUC57-neoAg with primers neoAg-F and neoAg-R. DTT-neoantigen fusion was generated by PCR with DTT and neoantigen fusion fragments as templates, DTT-F and neoAg-R as primers. The resulting recombinant DTT-neoAg fragment was double-digested with *BamH I* and *Xho I*, and cloned into pGEX-6p-1. DTT-wtAg construct was generated in the same way as described above except that the mutant residues are replaced with wild type residues. The neoantigen fragment was also fused to the C-terminal of CTB, the resulting recombinant protein CTB-neoAg was used as an ELISA coating antigen for detection of antibodies against neoantigens in mouse sera vaccinated with DTT-neoAg.

### Protein Expression and Purification

pGEX-DTT-neoAg and pGEX-DTT-wtAg were transformed into *E. coli* BL21(DE3), respectively. A single colony was inoculated into 3 mL LB media with 50 μg/mL ampicillin, cultured overnight at 37°C. The culture was expanded into 500 mL of LB media until OD600 nm reached 0.6. IPTG (isopropyl-ß-D-thiogalactoside) was then added to a final concentration of 0.5 mM. The culture was incubated at 16°C for 24 h. The cells were harvested by centrifugation, and the cell pellets were resuspended in phosphate-buffered saline (PBS) and lysed by sonication (60 cycles of 5 s on ice). The lysate was subject to centrifugation at 12 000 × g for 30 min at 4°C, and the supernatant was applied to GST affinity columns. GST tag was removed by PreScission protease cleavage at 4°C for 20 h, in 50 mM Tris-HCl, 140 mM NaCl, 1 mM EDTA, and 1 mM dithiothreitol, pH 7.4. The protein samples were analyzed by 15% ExpressPlus PAGE gels (GenScript, Nanjing, China).

### Mice Immunization

Female C57BL/6 mice (6–8 weeks of age, 5–10 per group) were injected subcutaneously into the lateral flank with 30 μg DTT-neoAg or DTT-wtAg, 300 μg Alum, and 30 μg CpG, formulated in 200 μL PBS. The control group of mice were administered with 300 μg Alum and 30 μg CpG. Each mouse received three injections at one- or 2-week intervals. Blood samples were drawn from orbital sinus 1 week after injection.

### ELISA for Antibody Detection

To detect antibodies against neoAg, ELISA plates were incubated overnight at 4°C with 100 ng CTB-neoAg, or DTT, or CTB-wtAg in 100 μL sodium carbonate buffer, pH 9.6. The non-specific binding sites were blocked with 100 μL 3% skim milk in PBS + 0.05% Tween 20 at room temperature for 1 h. Subsequently, the mouse sera with indicated dilution were added to the wells and incubated for 1 h at room temperature. The bound antibodies were detected using goat anti-mouse IgG-HRP, or goat anti-mouse IgG1-HRP, or IgG2a-HRP, or IgG2b-HRP, or IgG3-HRP, or IgM-HRP (1:5,000 dilutions, Shanghai Immune Biotech Co. Ltd., Shanghai, China) using 3,3′,5,5′-tetramethylbenzidine (TMB, TIANGEN, Shanghai, China) as substrate. The absorbance at 450 nm was measured by EnSpire 2300 ELISA reader (PerkinElmer, Waltham, MA, USA). The antibody titers are defined as the reciprocals of the highest dilution yielding an optical density of 0.2 or greater than that of pre-immune mouse sera.

### Tumor Challenge

For the prophylactic tumor model with DTT-neoantigen vaccination, C57BL/6 mice (*n* = 5–6, 6–8-weeks old) were subcutaneously immunized on day 0, 12, 24. 9 days after the third immunization, the mice were subcutaneously (s.c.) injected with 1 × 10^5^ B16F10 cells in 100 μL of PBS into the right flank. In prophylactic tumor model with neoantigen peptides (neoAg-pep) or DTT-wtAg vaccination, C57BL/6 mice (*n* = 6–8, 6–8-weeks old) were subcutaneously immunized on day 0, 10, 20. 7 days after the third immunization, the mice were subcutaneously (s.c.) injected with 1 × 10^5^ B16F10 cells in 100 μL of PBS into the right flank.

For the therapeutic tumor model, 6–8 weeks old C57BL/6 mice (*n* = 5–10) were first s.c. inoculated with 2.5 × 10^4^ B16F10 cells in 100 μL of PBS. Then they were administered with indicated vaccines 7 days after tumor cell injection and boosted twice at 1-week intervals. Animal appearance and behavior were monitored on a daily basis. Tumor sizes were measured every 2–3 days by calipers, and calculated using equation: volume = [(length) × (width)^2^]/2 in mm^3^. Ninety days after the first tumor challenge, the tumor-free mice were re-challenged s.c. with 7.5 × 10^4^ B16F10 cells on the left flank and monitored for tumor growth. Seven days after the rechallenge, the mice were injected s.c. with indicated vaccines three times at 1-week intervals. Mice were sacrificed when tumor volumes reached 2,000 mm^3^ and recorded as death.

### T Cells Proliferation and the Subset Analysis

Single cell suspensions were prepared with immunized mouse spleens and treated with ACK lysis buffer (0.15 mM NH_4_Cl, 10 mM KHCO_3_, 0.1 mM disodium EDTA, pH 7.2). The cells were plated in a 96-well flat plate at 1 × 10^5^ cells/well in 100 μL of RPMI-1640 medium supplemented with 10% FCS, and stimulated with 30 μg/mL CTB-neoAg protein or 5 μg/mL Con A. After 72 h, the culture was treated with CCK-8 solution following manufacture's instruction (YEASEN, Shanghai, China). The optical density (OD) of cells at wavelength 450 nm was measured with OD at 650 nm as a reference. Stimulation index (SI) was calculated as the ratio of optical density (OD) 450 nm of stimulated cells to that of unstimulated cells.

To detect CD8^+^ T cells, freshly isolated spleen lymphocytes were stimulated with 30 μg/mL CTB-neoAg protein for 72 h. Unstimulated cells were used as negative control. The lymphocytes were stained with anti-mouse CD3ε antibody-FITC and anti-mouse CD8 antibody-PerCP-Cy5.5, and analyzed using a FACS Calibur instrument (Beckman Coulter, USA).

For CD19^+^ B cell analysis, the freshly isolated spleen lymphocytes were stained with anti-mouse CD19 antibody-FITC. To measure Fop3^+^/CD4^+^ ratio, tumor-infiltrating lymphocytes or spleen lymphocytes were isolated from DTT-neoAg-treated mice or PBS-treated mice and stained with anti-mouse CD3ε antibody-APC (BD Biosciences) and CD4 antibody-FITC (BD Biosciences). Tumor infiltrating leucocytes were prepared from subcutaneous B16F10 tumors when tumor volumes reached 1500 mm^3^. Tumor tissues were ground and filtered through a 70-μm cell strainer. The TILs are purified by a tumor infiltrating lymphocytes separation solution according to the manufacturer's protocol (Beijing Solarbio Science and Technology Co., Beijing, China). Then intracellular FoxP3^+^ staining was performed according to the manufacturer's protocol (Mouse Foxp3 Buffer Set, BD). The samples were analyzed using a FACS Calibur instrument (Beckman Coulter, USA). All experiments were repeated three times and the average values were calculated.

### Intracellular Cytokine Staining

The mutation-specific IFN-γ^+^ T cells were detected by intracellular cytokine staining (ICS), and analyzed by flow cytometry. Bone marrow-derived dendritic cells (BMDCs) were obtained by culturing bone marrow cells of naïve C57BL/6 mice in RPMI-1640 medium containing 10% FBS, GM-CSF (20 ng/mL) and IL-4 (20 ng/mL) (Sino Biological, Beijing, China) for 6 days as previously described (26). BMDCs were loaded with or without 50 μg/mL DTT-neoAg overnight.

Splenocytes harvested from DTT-neoAg-immunized mice were incubated for 5 min at room temperature in ACK Lysing Buffer (0.15 mM NH_4_Cl, 10 mM KHCO_3_, 0.1 mM disodium EDTA, pH 7.2) and then washed in RPMI-1640 medium (Gibco-BRL) with 10% fetal bovine serum (FBS). The splenocytes (3 × 10^6^) were co-incubated with DTT-neoAg-loaded BMDCs (3 × 10^5^) for 48 h while splenocytes co-incubated with BMDCs served as a negative control. Cells were further incubated for 6 h at 37°C in the presence of Brefeldin A (3.0 μg/mL, Sino Biological, Beijing, China). The splenocytes were treated with phorbol 12-myristate 13-acetate (PMA, 81 nM) and Ionomycin (1.34 μM, Thermo Fisher Scientific) served as a positive control. The cells were then harvested and stained with anti-CD3ε antibody-PerCP-Cy 5.5 (BD Biosciences), anti-CD4 antibody-FITC (BD Biosciences), and anti-CD8 antibody-PE (BD Biosciences) followed by anti-IFN-γ antibody-APC (BD Biosciences) staining after Cytofix/Cytoperm treatment according to the manufacturer's protocol (BD Biosciences). Subsequently, the samples were analyzed on a CytoFLEX flow cytometer (Beckman Coulter), and the data were analyzed using FlowJo software.

### Cytotoxicity Assay

Cytotoxic T lymphocyte assay was performed as previously described (27). Briefly, spleen lymphocytes or TILs stimulated with 30 μg/mL CTB-neoAg protein for 72 h in the presence of 20 units/mL rhIL-2 (ExCell Bio, Shanghai, China) were used as effector cells. The stimulated lymphocytes were co-cultured with B16F10 (target cells) at different effector to target ratios (50:1, 20:1, and 10:1) for 4 h at 37°C. Fifty microliter of the culture supernatant was used to assess cytotoxic activity by CytoTox96 non-radioactive cytotoxicity assay kit (Promega, Madison, WI, USA) according to the manufacturer's instructions.

### IFN-γ Assay

Splenocytes were isolated from immunized mice, stimulated with 30 μg/mL CTB-neoAg in 24-well culture plates (5 × 10^5^/well, in 500 μL media) at 37°C with 5% CO_2_. The culture media were collected at indicated time points. The IFN-γ concentrations were measured by ELISA kit (R&D Systems, USA).

### Quantitative PCR Assay

Total tumor RNA were prepared using TRIzol reagent (Invitrogen). cDNA were reverse-transcribed using PrimeScript™ RT reagent Kit with gDNA Eraser (Takara, Dalian, China). Real-time PCR was performed using the TB Green™ Premix Ex Taq™ II according to the manufacture's protocol (Takara, Dalian, China). The mRNA levels of IFN-γ, IL-12, IL-4, and IL-10 in each sample were calculated using the 2^−ΔΔ*ct*^ method. The primer sequences are listed in Supplementary Table 2.

### Immunohistochemistry Analysis

Immunohistochemistry was performed as described previously (8). Tumor tissues, livers, kidneys, or lungs were fixed in 4% paraformaldehyde and embedded in paraffin. The paraffin-embedded tissues were cut into 4 μm-slides, stained with hematoxylin and eosin (H&E) or rat anti-mouse CD8 antibody (Santa Cruz Biotechnology Inc., Dallas, TX, USA) or anti-mouse NK1.1 antibody (Bio Legend, USA) according to the manufacture's protocol. Immunohistochemical detection was performed following the instruction of MaxVision^TM^ kit (Fuzhou Maixin Biological Inc., Fuzhou, China). Five different fields for each slice were selected under 200 × magnification to quantify the percentages of CD8^+^ cells by Image J.

### Statistical Analyses

All data were analyzed using GraphPad Prism 7.0 software and were presented as mean ± standard deviation (SD). The statistical significance between two groups was analyzed by a two-tailed, unpaired Student's *T*-test. The survival curves were generated using the Kaplan-Meier method and the statistical significance between two groups were analyzed using the log-rank test. ^*^indicates *P* < 0.05, ^**^indicates *P* < 0.01, ^***^indicates *P* < 0.001, ^****^indicates *P* < 0.0001, and ns indicates no significant difference.

## Result

### The Design of Vaccine Immunogen DTT-neoAg

Activation of effector CD4^+^ T cells is a hallmark of sustained and protective immunity induced by vaccination (28). For neoantigen-based cancer vaccine, antigen-specific CD4^+^ T cell effectors can lead to tumor regression through direct cytotoxic mechanisms (29) or activation of macrophages (30). Due to immune tolerance, self-molecule proteins are not recognized by T cell receptors under normal physiological conditions (31). This self-tolerance can be bleached by insertion or fusion of a foreign helper T cell epitope into the self-molecules (32). In our previous studies, we found insertion of a neutralizing epitope peptide of TNF α or EGFR into a membrane translocation domain of Diphtheria toxin, named DTT, can induce TNFα- or EGFR-specific antibody responses by virtual of its several universal Th epitopes (25, 33). When formulated with Th1-inducing adjuvants such as poly I/C or CpG, EGFR-specific cytotoxic T lymphocytes were activated which conferred antitumor immunity in mouse tumor models (33). Moreover, fusion of self-protein domains to DTT also induced antibody responses toward self-proteins such as EGFR, TNF α, VEGF, FXI (24, 25, 33, 34). In this study, we applied DTT fusion strategy to neoantigen-based vaccine design, and tested the immunogenicity of those tumor neoantigens predicted *in silico* to possess good affinity to MHC I molecules, but experimentally failed to elicit any tumor-specific cytotoxic T cell responses.

**Figure 1:**
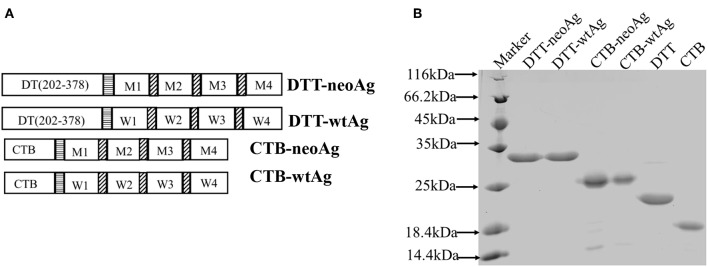
Design and expression of DTT adjuvant-based neoantigen vaccine. **(A)** A schematic diagram of DTT-neoAg and DTT-wtAg. M1, M2, M3, and M4 are the mutation peptide of Pi4k2b, Ddb1, Pcdhga11 and Atp11a, respectively. W1, W2, W3, and W4 are the corresponding wild type peptides. Each mutation peptide is 27 amino acid residues in length with the mutated residue positioned in the central (position 14). The horizontal stripe box stands for the linker sequence (GGGGSGGGGS) and the diagonal stripe box stands for the linker sequence (GS). **(B)** 15% SDS-PAGE analysis of the purified recombinant proteins.

We chose four MHC I-binding, non-immunogenic mutation peptides identified in a previous report by Sahin (7) as a part of vaccine immunogen. These neoantigen peptides are encoded by genes of Pi4k2b, Ddb1, Pcdhga11, Atp11a, and the corresponding mutations are verified in our laboratory B16F10 cell line. Indeed, these 27aa neoantigen peptides failed to elicit anti-tumor immune response when formulated with Alum and CpG (Figures S1B,C).The mutation peptides are linked together by GS linkers (7) and fused to the C-terminal of DTT as shown in Figure 1A. The recombinant immunogen is named DTT-neoAg.

An *E. coli* expression vector was constructed for DTT-NeoAg. For control experiments, an expression vector of corresponding wild-type peptide antigens, named as DTT-wtAg, was also constructed. The sequences of neoantigens and their wild-types were provided in the Supplementary Table 3. For detection of neoAg-specific antibody responses by ELISA and *in vitro* expansion of neoAg-specific T cells, an expression vector was constructed for neoAg fused to C-terminal of Cholera Toxin B chain, named as CTB-neoAg.

The DTT-neoAg and DTT-wtAg were expressed in *E. coli* as soluble GST-tagged recombinant proteins. After GST-affinty purification, the GST tag was removed by PSP cleavage and GST affinity column chromatography (Figure 1B).

### DTT-neoAg Induces Neoantigen-Specific Antibody and Antitumor Cellular Immune Responses in Mouse

**Figure 2:**
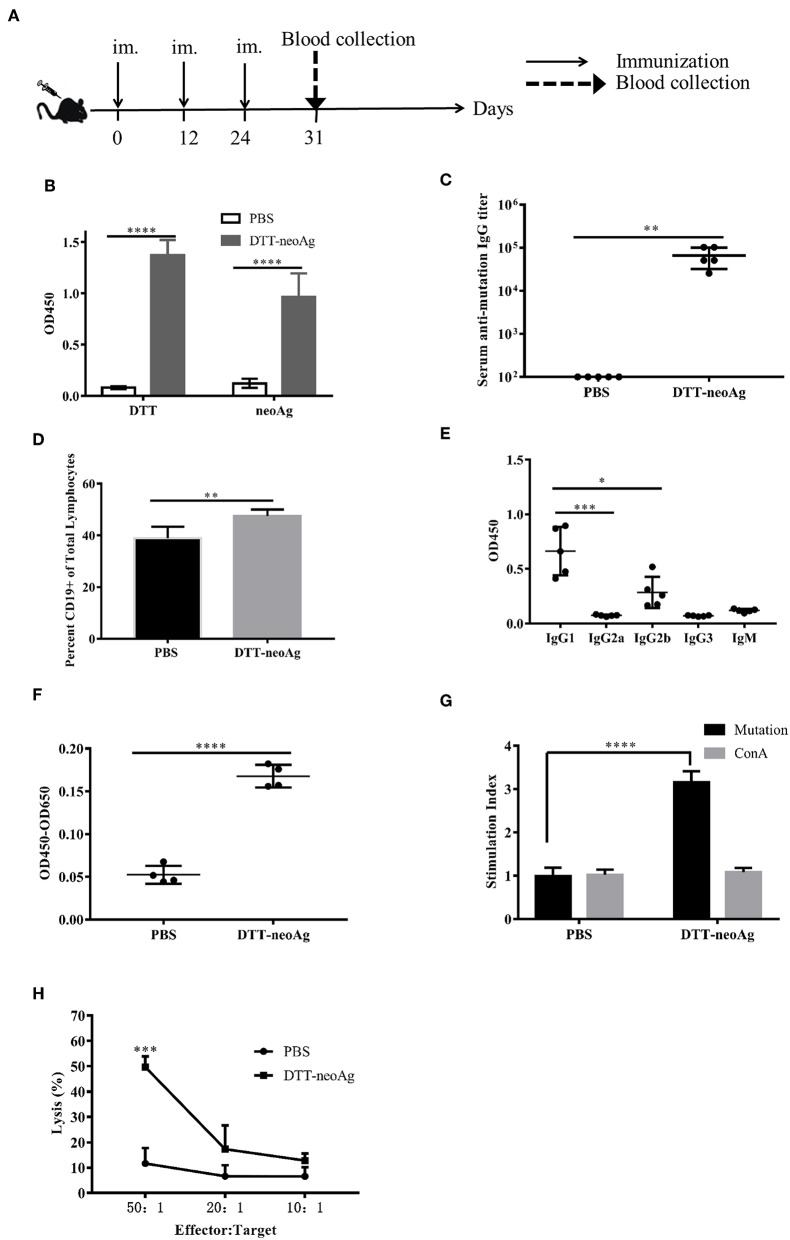
DTT-neoAg induces neoantigen-specific antibodies and antitumor cellular immune responses in mouse. The statistical significances were determined by Student's T test. ^****^P < 0.0001; ^***^P < 0.001; ^**^P < 0.01; ^*^P < 0.05. **(A)** A schematic diagram of the experimental design. **(B–D)** Female C57BL/6 mice (*n* = 5) were immunized subcutaneously on day 0, 12, 24 with DTT-neoAg vaccine. One week after the third immunization, mouse sera were collected by orbital blood sampling, and the antibody responses were analyzed by ELISA. **(B)** ELISA with sera in 1:100 dilution and with indicated coating antigens. **(C)** The antibody titers against the neoantigen. **(D)** The percentage of CD19^+^ B cells in the lymphocytes of immunized mice. Seven days after the third immunization, the splenocytes of the vaccinated mice were stained with anti-CD19-FITC, and the percentage of CD19^+^ B cells in the lymphocytes was analyzed by flow cytometry. **(E)** The antibody subclass analyses. The sera were 1:100 diluted. **(F–H)** DTT-neoAg vaccine induced cellular immune response. Six to eight weeks old female C57BL/6 mice (*n* = 3) were immunized with DTT-neoAg or PBS formulated with Alum + CpG, respectively, at 2-week intervals. Seven days after the third immunization, the splenocytes were stimulated with CTB-neoAg or PBS for 72 h. Cell proliferation **(F)** and stimulation indices **(G)** were measured by CCK8 kit. **(H)** The splenocytes from DTT-neoAg-treated mice or PBS-treated mice were stimulated with CTB-neoAg (30 μg/mL) for 72 h, and were used as effector cells, then co-cultured with target cells B16F10 at indicated ratios for 4 h at 37°C. The percentage of cell lysis was determined by LDH assay.

We immunized C57BL/6 mice with DTT-neoAg formulated with Alum adjuvant and CpG (Figure 2A). The control mice were administered with PBS + Alum + CpG. One week after the third injection, ELISA was performed to measure the antibody responses against neoAg. We observed robust antibody responses in DTT-neoAg treated mice (Figure 2B), but not in PBS treated mice, with the antibody titers at the levels of 6 × 10^5^ (Figure 2C).

Because antibody responses depend on B cells, we tested whether the vaccine can increase the number of B lymphocytes in spleen. Seven days after the third immunization, the splenocytes of DTT-neoAg or PBS-treated mice were labeled with CD19 antibody, and analyzed by flow cytometry. We found that the percentage of CD19^+^ lymphocytes in DTT-neoAg-treated mice was 47.47 ± 1.04%, while in the PBS-treated mice was 38.87 ± 1.82% (Figure 2D). Therefore, the B cells of DTT-neoAg-immunized mice were increased significantly in comparison with those of the PBS group mice.

We further analyzed the antibody subclasses in DTT-neoAg-treated mouse sera (Figure 2E), and found that the subclasses of IgGs were primarily IgG1 and IgG2b. The other subclasses including IgM, IgG2a, and IgG3 were at below detection levels. As the ratio of IgG1/IgG2b was >1, we conclude that immune responses induced by DTT-NeoAg is biased toward Th2 type.

To examine the cellular immune responses of DTT-neoAg vaccination, we stimulated the splenocytes of each group of mice with CTB-neoAg *in vitro* for 72 h, and measured the cell proliferation by CCK8 kit. Significant cell proliferation was observed in the splenocytes of DTT-neoAg-treated mice (Figure 2F) with the average stimulation index 3 times that of PBS-treated ones (Figure 2G). The cell proliferation was similar when both groups of the splenocytes were stimulated with Con A (Figure 2G). These data indicated that DTT-neoAg vaccine can induce mutation-specific T cell memory responses.

We next evaluated the cytotoxicity of DTT-neoAg-treated splenocytes by LDH release assay after *in vitro* stimulation with CTB-neoAg. At effector-target ratios of 50:1 and 20:1, the percentages of cell lysis were 49.65 ± 4.19 and 17.29 ± 9.33%, respectively (Figure 2H). For the splenocytes of PBS-treated mice, the percentages of cell lysis were 11.62 ± 6.06 and 6.64 ± 4.33%, respectively. These data indicated that DTT-neoAg vaccination elicit robust cytotoxic immune responses against B16F10.

### DTT-neoAg Vaccination Significantly Inhibits Tumor Growth in a Prophylactic Mouse Tumor Model

**Figure 3:**
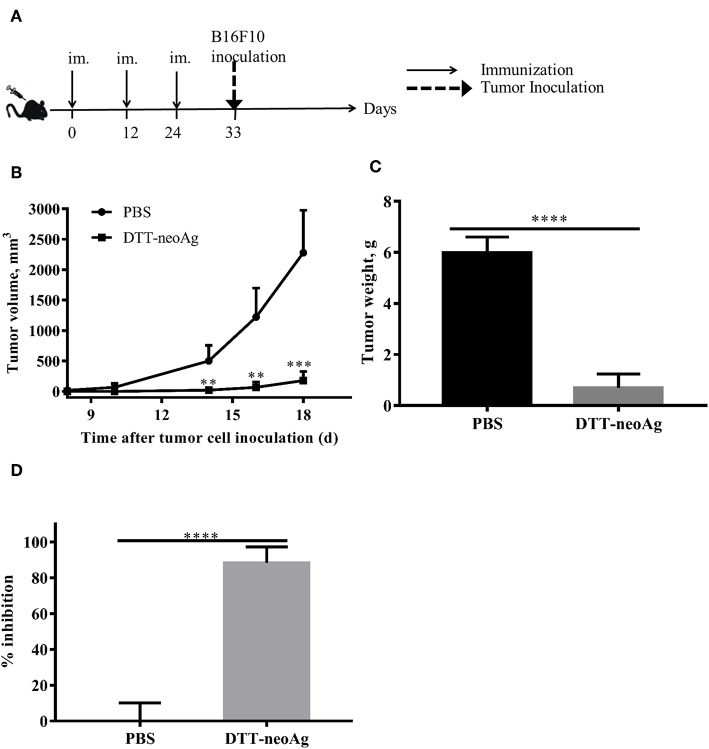
Anti-tumor effects of DTT-neoAg vaccination in the prophylactic mouse melanoma model. The data are means ± SEM, ^****^P < 0.0001; ^***^P < 0.001; ^**^P < 0.01; ^*^P < 0.05, Student's T-test. **(A)** The treatment schedule. C57BL/6 mice (*n* = 5) were immunized with DTT-neoAg or PBS formulated with Alum + CpG on day 0, day 12 and 24. Nine days after the third immunization, 1 × 10^5^ B16 F10 cells were s.c. administered into the right flank of the mice. **(B)** The tumor growth curves. **(C)** On day 22 after the tumor challenge, tumors weights were measured. **(D)** Tumor inhibition rate of PBS-treated or DTT-neoAg-treated mice.

Further to assess whether the vaccine-elicited immune responses render tumor growth inhibition, we challenged the vaccinated mice subcutaneously with 1 × 10^5^ B16F10 in the right flank 9 days after the third immunization (Figure 3A). While tumors were palpable in all of mice treated with PBS + adjuvant on day 10 after tumor challenge, the DTT-neoAg-treated mice were all tumor-free. Tumors were detected in 4 of 6 mice treated with DTT-neoAg on day 16 after the tumor challenge. More strikingly, one DTT-neoAg-treated mouse was tumor-free until 30 days after tumor inoculation. Furthermore, the tumors in DTT-neoAg-treated mice grew much slower than those of PBS-treated ones. On day 18, the average tumor volumes of PBS-treated mice was 2278 ± 349.2 mm^3^ while that of DTT-neoAg was 177.7 ± 66.53 mm^3^, a growth reduction by more than 80% (Figure 3B). On day 22, the average tumor weight was 0.7 ± 0.27 g for DTT-neoAg treated-mice in contrast with that of PBS-treated ones which was 6 ± 0.27 g (Figure 3C). Therefore, DTT-neoAg vaccine reduced the tumor growth by 88% (Figure 3C).

In order to prove that DTT is necessary to elicit the immune response against neoAg, and only an immune response specific to neoAg can inhibit tumor, we s.c. injected 1 × 10^5^ B16F10 in the right flank of mice, 7 days after the third immunization with neoAg-pep + adjuvant or DTT-wtAg + adjuvant, or PBS + adjuvant (Figure S1A). Seven days after the challenge, tumors were detected in all of the PBS-treated mice, neoAg-pep-treated mice and DTT-wtAg-treated mice. Meanwhile, the tumor volumes of PBS-treated mice, neoAg-pep-treated mice and DTT-wtAg-treated mice were 12.2 ± 2.74, 11.31± 3.24, and 16.81 ± 2.97 mm^3^, respectively. On day 17, the average tumor volumes of PBS group, neoAg-pep group, and DTT-wtAg were 1801 ± 378.8, 1514 ± 251.2, and 1439 ± 133.6 mm^3^, respectively. Overall, there was no significant difference in the growth curves of each group (Figure S1B). The median survival rates of the three groups were all 19 days (Figure S1C). Therefore, both neoAg-pep and DTT-wtAg failed to inhibit tumor growth in the prophylactic mouse tumor model.

### DTT-neoAg Vaccination Inhibits Tumor Growth in the Therapeutic Mouse Tumor Model

**Figure 4:**
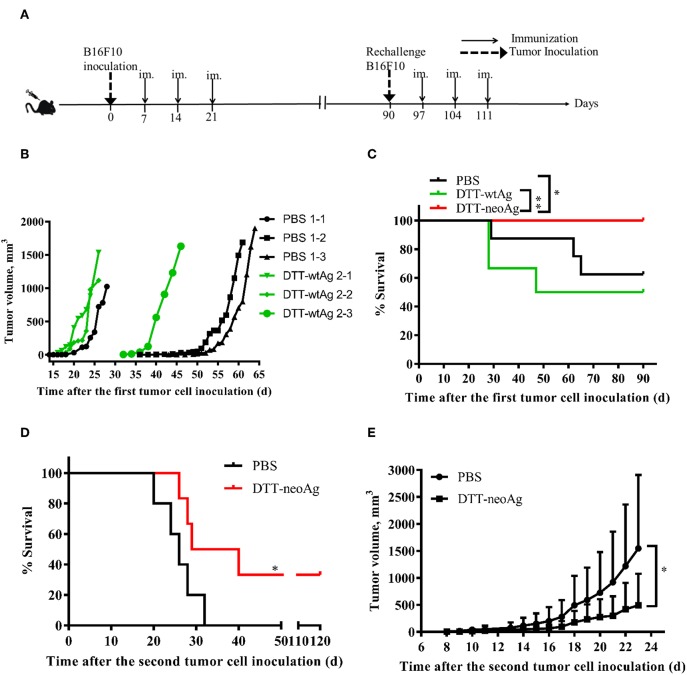
Anti-tumor effects of DTT-neoAg vaccination in the therapeutic mouse melanoma model. The data are means ± SEM, ^****^P < 0.0001; ^***^P < 0.001; ^**^P < 0.01; ^*^P < 0.05, Student's T-test. **(A)** The treatment schedule. **(B,C)** C57BL/6 mice (*n* = 6–13) were s.c. administered with 2.5 × 10^4^ B16F10 cells into the right flank of the mice. Seven days after challenge, mice were immunized with DTT-neoAg, DTT-wtAg, or PBS formulated with Alum + CpG three times at 1-week intervals. **(B)** The tumor growth curves for individual mouse. Green line: DTT-wtAg-treated mice, and black line: PBS-treated mice. **(C)** The Kaplan-Meier survival plot. The statistical significance was determined by Log-rank test. ^*^P < 0.05. **(D,E)** On day 90, the tumor-free mice in PBS-treated group and neoAg-treated group were re-challenged with 7.5 × 10^4^ B16F10 cells in the left flank. One week after the re-challenge, mice were immunized with PBS or neoAg formulated with Alum + CpG for 3 times at weekly intervals. **(D)** The Kaplan-Meier survival plot. The statistical significance was determined by Log-rank test. ^*^P < 0.05. **(E)** The tumor growth curves.

B16F10 melanoma cell line is highly aggressive when implanted into a syngeneic host (35). Therefore, to examine therapeutic efficacy of DTT-neoAg vaccine, we s.c. injected a low dose of 2.5 × 10^4^ B16F10 cells in the right flanks of mice to allow sufficient time for the vaccine to induce anti-tumor immunity. Seven days after the tumor challenge, mice were administered with the indicated vaccines and boosted twice at 1 week intervals (Figure 4A). Tumors were detected in some of the PBS-treated mice as early as 18 days after tumor challenge (Figure 4B). On day 43, 37.8% of PBS-treated mice developed tumors. Tumors were detected in 16.7% of DTT-wtAg-treated mice on 15 days, 3 days earlier than those of PBS group of mice (Figure 4B), and on day 32, 50% of the mice had tumors. In contrast, all DTT-neoAg-treated mice were tumor-free on day 90, and the overall survival of the DTT-neoAg-immunized group was significantly prolonged when compared to two control groups. The survival rates for PBS and DTT-wtAg groups were similar (Figure 4C). Moreover, mice of the control groups appeared weak, unresponsive, less active, and arched since day 14 while DTT-neoAg-treated mice behaved normal. These data suggest that DTT-neoAg induce therapeutic and mutation-specific antitumor immunity.

We next challenged the tumor-free mice with a higher dose of B16F10 cells to evaluate therapeutic efficacy of DTT-neoAg vaccine. The tumor-free, PBS-treated or DTT-neoAg-treated mice were challenged with 7.5 × 10^4^ B16F10 cells on the left flanks on day 90. These mice were vaccinated three more times 7 days after the second tumor challenge as indicated in Figure 4A. Strikingly, 33.33% of the mice treated with DTT-neoAg were tumor-free even on day 120, whereas all the mice in the PBS-treated group developed tumors, and died within 32 days (Figure 4D). The average survival days of the tumor-bearing, DTT-neoAg-treated mice was 34.5, which is 8.5 days longer than that of PBS-treated group (Figure 4D). Consistently, the tumor growth was markedly reduced in DTT-neoAg-immunized mice when compared with that of PBS-immunized mice (Figure 4E). Taken together, these data indicated that DTT-neoAg vaccine confer therapeutic benefit to tumor bearing mice.

### DTT-neoAg Vaccination Induces Humoral and Cellular Immune Responses in Tumor Challenged Mice

**Figure 5:**
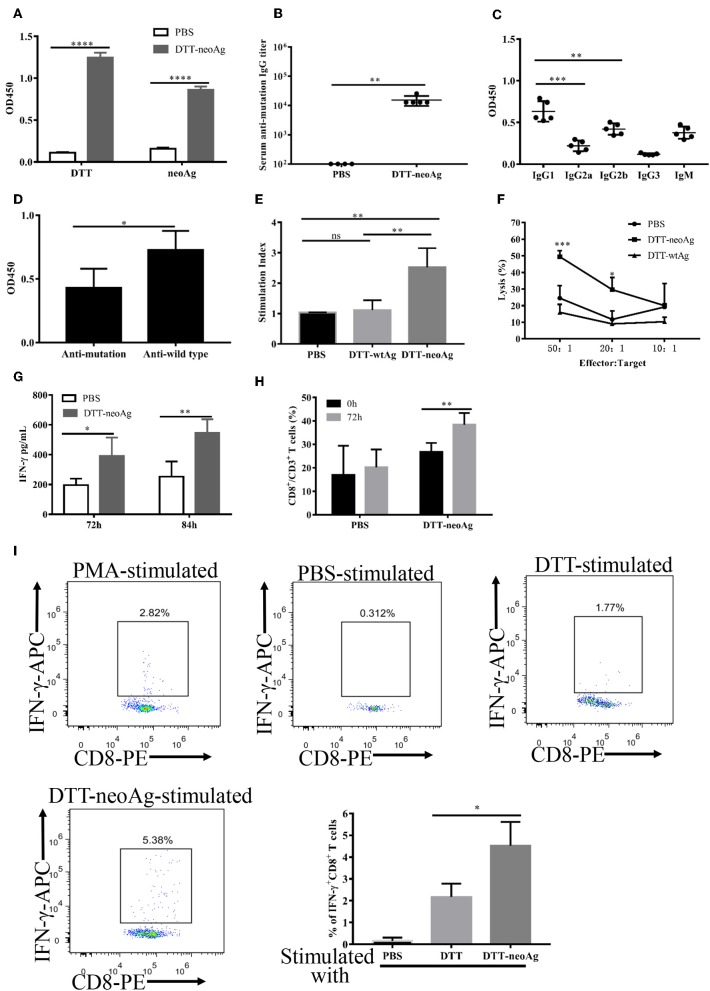
Antibody responses and cellular immune responses elicited by DTT-neoAg vaccination in tumor challenged mice. The statistical significance was determined by Student's T-test. ^***^P < 0.001; ^**^P < 0.01; ^*^P < 0.05. **(A–D)** C57BL/6 mice (*n* = 6–13) were administered s.c. with 2.5 × 10^4^ B16 F10 cells into the right flank of the mice. Seven days after tumor challenge, mice were immunized three times at weekly intervals with DTT-neoAg, DTT-wtAg, or PBS, formulated with Alum + CpG. **(A)** The mouse sera were collected on day 7 after the third immunization. The antibody responses were measured by ELISA with the sera 1:100 diluted and with the indicated coating antigens. **(B)** The anti-neoAg antibody titers were measured by ELISA. **(C)** Analysis of anti-neoAg antibody subclasses. **(D)** Anti-sera from DTT-wtAg-treated mice crossreact with neoAg. The sera were collected from mice immunized with DTT-wtAg after the third immunization, and the antibody specificity was analyzed by ELISA with sera 1:100 diluted and CTB-wtAg or CTB-neoAg as a coating antigen. **(E)** The splenocytes from indicated mice were stimulated *in vitro* with CTB-neoAg for 3 days. The stimulation indices was measured by CCK8 assay. **(F)** The cytotoxic T lymphocytes induced by DTT-neoAg vaccination. The splenocytes from DTT-neoAg-treated, or DTT-wtAg-treated, or PBS-treated mice were stimulated with CTB-neoAg for 3 days, and used as effector cells. B16F10 cells were used as target cells. The splenocytes and B16F10 cells were co-cultured at indicated ratios for 4 h at 37°C. The percentage of cell lysis was measured by LDH assay. Asterisks indicate statistically significant differences between DTT-neoAg-treated and DTT-wtAg-treated mice. **(G)** INF-γ production by *in vitro* neoAg-stimulated splenocytes of DTT-neoAg vaccinated mice. The splenocytes were stimulated with CTB-neoAg for 72 or 84 h, and the INF-γ in the culture supernatant was analyzed using a mouse IFN-γ ELISA kit. **(H)** CD8^+^ memory T cells induced by DTT-neoAg vaccination. The splenocytes of the vaccinated mice were *in vitro* stimulated with CTB-neoAg for 72 h, stained with anti-CD3ε-FITC anti-CD8-PerCP-Cy5.5, and the percentage of CD8^+^ T cells in CD3^+^ T cells was analyzed by flow cytometry. **(I)** The splenocytes of DTT-neoAg-treated mice were incubated with antigen-loaded DC for 48 h, then Golgi-stop was added; 6 h later, the cells were harvested and stained with anti-CD3ε-PerCP-Cy5.5, anti-CD4- FITC, anti-CD8-PE Ab. After permeabilization, intracellular cytokines were stained with anti-IFN-γ-APC antibody and analyzed by flow cytometry.

Since DTT-neoAg vaccination provided efficient tumor control in the therapeutic tumor model, we assessed the antibody responses and cellular immune responses in tumor bearing mice elicited by the vaccine. We measured antibodies against DTT and neoAg by ELISA, and found that both anti-DTT and anti-neoAg antibodies were robust with antibody titers up to 1.5 × 10^5^ (Figures 5A,B). As the ratios of IgG1 to IgG2a were >1, the induced immunity was biased toward Th2 type (Figure 5C). Compared to the sera from mice treated with DTT-neoAg before the tumor challenge, more IgG2a (12% vs. 6%) and less IgG1 (37% vs. 54%) were generated when DTT-neoAg vaccine was applied after the tumor challenge, suggesting that immune response shifted toward Th1 type of immunity in therapeutic setting (Figure S2A). However, the class switch was not complete in mice of the therapeutic tumor model as the IgM subclass was present in the mouse sera.

In mice treated with DTT-wtAg vaccine, robust antibody responses against wtAg were observed with weak cross activities to neoAg (Figure 5D). This result is consistent with a T7 phage-based vaccine targeting multiple neoepitopes including Atp11a mutations (36). Since DTT-wtAg vaccination failed to inhibit tumor growth, the antitumor immunity induced by DTT-neoAg was mutation-specific.

We then evaluated the cellular immune responses induced by DTT-neoAg vaccine. Thus, the splenocytes were stimulated with CTB-neoAg for 72 h, the cell proliferations as well as the cytotoxic activities were measured. As shown in Figure 5E, significant cell proliferations were observed only in splenocytes of DTT-neoAg-treated mice. The stimulation index was almost 3 times that of DTT-wtAg or PBS treated mice, which indicated that DTT-neoAg induced mutation-specific cellular responses. Again, significant cytotoxic activities were observed only in DTT-neoAg treated mice (Figure 5F). The percentage of neoAg-specific cell lysis was 49.46 ± 2.07% at effector/target ratio of 50:1, and 29.58 ± 4.27% at lower ratio of 20:1 (Figure 5F).

Further to examine whether the cytotoxic activities were conferred by CD8^+^ T cells, we first measured the amount of INF-γ in the culture media of the splenocytes stimulated with CTB-neoAg, and found that DTT-neoAg-vaccinated mice splenocytes secret twice amount of INF-γ as compared to that of PBS treated mice splenocytes (Figure 5G). In addition, the percentage of CD8^+^ CD3^+^ lymphocytes in DTT-neoAg-treated mice was increased from 26.8 ± 3.81% to 38.42 ± 4.94% by CTB-neoAg stimulation (Figure 5H). Next, we labeled the splenocytes of DTT-neoAg-treated mice stimulated *in vitro* by DTT-neoAg with CD8 antibodies, and found that ~4.5% of the CD8^+^ T cell population expressed IFN-γ, which was 2-fold higher than that of the same splenocytes stimulated with control DTT (Figure 5I), indicating that neoantigen-specific CTLs were induced by the vaccine. In contrast with RNA and long-peptide neoantigen vaccines in preclinical and early phase clinical studies showing that immune responses were predominantly of CD4^+^ T cells (7, 8), DTT fusion strategy significantly enhanced immunogenicity of neoantigens as well as the CTL responses.

### DTT-neoAg Vaccination Enhances Intra-tumor Th1 Immunity and Causes Tumor Necrosis

**Figure 6:**
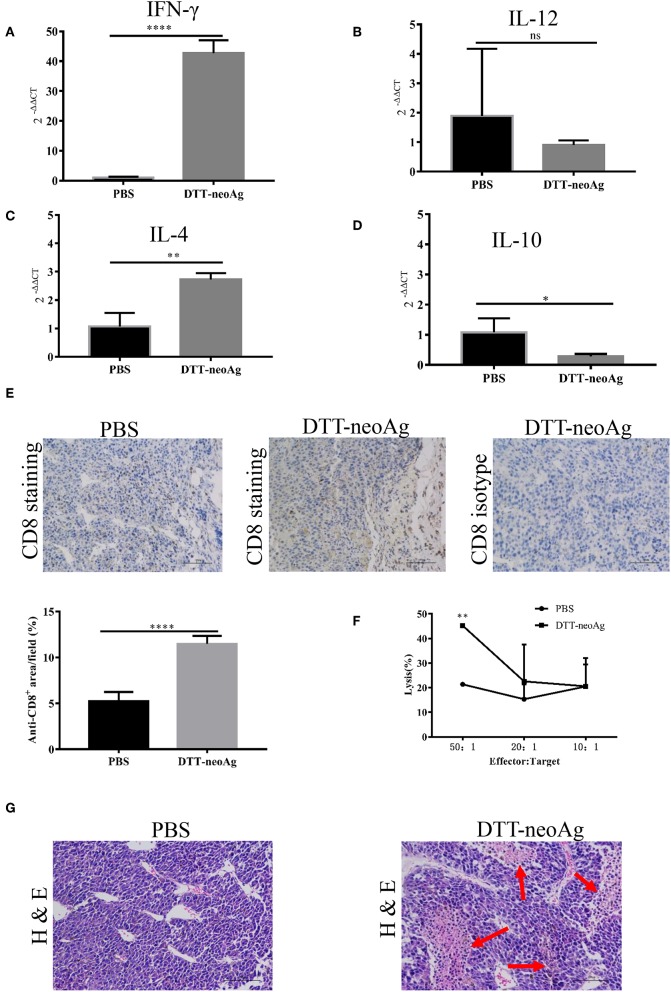
DTT-neoAg vaccination increases tumor necrosis and enhances CD8^+^ T cells infiltration. The statistical significances were determined by Student's T-test. ^****^P < 0.0001; ^***^P < 0.001; ^**^P < 0.01; ^*^P < 0.05. Frozen sections of B16F10 tumor tissues isolated from mice immunized with DTT-neoAg or PBS as in Figure 3D. **(A–D)** The relative expression levels of IFN-γ, IL-12, IL-4, IL-10 in the tumor tissues were determined by RT-PCR, respectively. **(E,G)** B16F10 tumors from indicated mice (*n* = 3) were collected and sectioned when the volumes reached 2,000 mm^3^. **(E)** The tissues sections were stained with anti-CD8 antibody. The density of CD8^+^ T cells were quantified by Image J. Numbers in the panel indicate average values of three samples per group, quantified by Image J. **(F)** The TILs from DTT-neoAg-treated mice or PBS-treated mice were stimulated with CTB-neoAg (30 μg/mL) for 72 h, and were used as effector cells, then co-cultured with target cells B16F10 at indicated ratios for 4 h at 37°C. The percentage of cell lysis was determined by LDH assay. **(G)** H&E staining of B16F10 tumor sections from indicated mice. Red arrows indicate the necrosis area, scale bar length is 100 μm.

To characterize the immune responses in the tumor tissues, we measured the cytokine levels by RT-PCR and CD8^+^ T cell infiltration by immunohistochemistry analyses. As shown in Figures 6A–D, tumor tissues from DTT-neoAg-treated mice expressed markedly higher levels of IFN-γ and IL-4 than PBS-treated mice while the levels of IL-12 and IL-10 were similar. The levels of INF-γ in DTT-neoAg-treated mice were increased by 40-fold in comparison with those of PBS-treated mice (Figure 6A), and the levels of IL-4 were increased by 2.7-fold (Figure 6C). Both IL-12 (Figure 6B) and IL-10 (Figure 6D) were detected, although the levels were low and show no significant difference between the experimental groups.

Furthermore, the percentage of CD8^+^ T cells in the tumor lesions of DTT-neoAg immunized mice was 11.48 ± 0.35%, a 2-fold increase as compared with that of the PBS-treated mice (5.25 ± 0.41%) (Figure 6E).

To verify whether tumor-infiltrating lymphocytes have anti-tumor cytotoxic activity, we isolated TILs from tumor tissues of DTT-neoAg-treated or PBS-treated mice. The TILs were stimulated with CTB-neoAg for 72 h, and the cytotoxic activities were measured. Indeed, significant cytotoxic activities were observed in TILs of DTT-neoAg-treated mice. The percentage of neoAg-specific cell lysis was 45.21 ± 0.67% at effector/target ratio of 50:1, but of the PBS group was only 21.37 ± 0.61% at ratio of 50:1 (Figure 6F). In addition, extensive necrosis and hemorrhage were observed in tumor sections of DTT-neoAg-immunized mice (Figure 6G), but not in those of the PBS-treated mice. These data indicated that DTT-neoAg induced a mix of Th1 and Th2 immunity in tumors.

### DTT-neoAg Vaccination Promotes NK Cells Infiltration into Tumor and Reduces Foxp3^+^/CD4^+^ Ratio in TILs and Spleens

**Figure 7:**
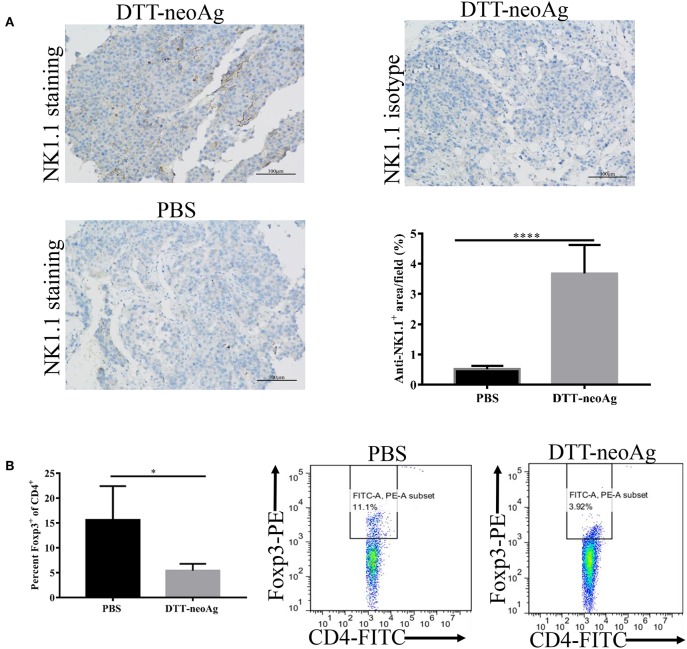
DTT-neoAg vaccination enhances NK1.1^+^ cell infiltration and reduces Foxp3^+^/CD4^+^ ratio in TILs and spleens. The statistical significances were determined by Student's T-test. ^****^P < 0.0001; ^***^P < 0.001; ^**^P < 0.01; ^*^P < 0.05. **(A)** B16F10 tumors from indicated mice (*n* = 3) were collected and sectioned when the tumor volumes reached 1,500 mm^3^. The tissues sections were stained with anti-NK1.1 antibody. The density of NK1.1^+^ cells were quantified by Image J. Numbers in the panel indicate average values of three samples per group, quantified by Image J. **(B)** The splenocytes from DTT-neoAg or PBS vaccinated mice were isolated and stained with anti-CD3ε-APC, anti-CD4- FITC. After permeabilization, cells were stained with anti-Foxp3-PE antibody and analyzed by flow cytometry.

Further to assess the effect of the DTT-neoAg vaccination on cellular immune composition of TILs, we measured the percentage of NK cells in the tumor tissues and FoxP3^+^/CD4^+^ ratio in the TILs. The percentage of NK1.1^+^ cells in the tumor lesions of DTT-neoAg immunized mice was 3.67 ± 0.38%, a 7-fold increase as compared with that of the PBS-treated mice (0.51 ± 0.04%) (Figure 7A).

The FoxP3^+^/CD4^+^ ratio in the TILs of DTT-neoAg-vaccinated mice was lower than that of PBS-treated mice (Figure S3A), although they were statistically not significant. The ratio of Foxp3^+^/CD4^+^ (5.43 ± 0.59%) in the spleens of mice treated with DTT-neoAg was markedly decreased as compared to that of PBS-treated mice (15.62 ± 3.39%) (Figure 7B). These data indicate that DTT-neoAg vaccine can alter cellular immune composition of the tumor microenvironments as well as the spleens.

### Toxicity Evaluation of DTT-neoAg Vaccine

**Figure 8:**
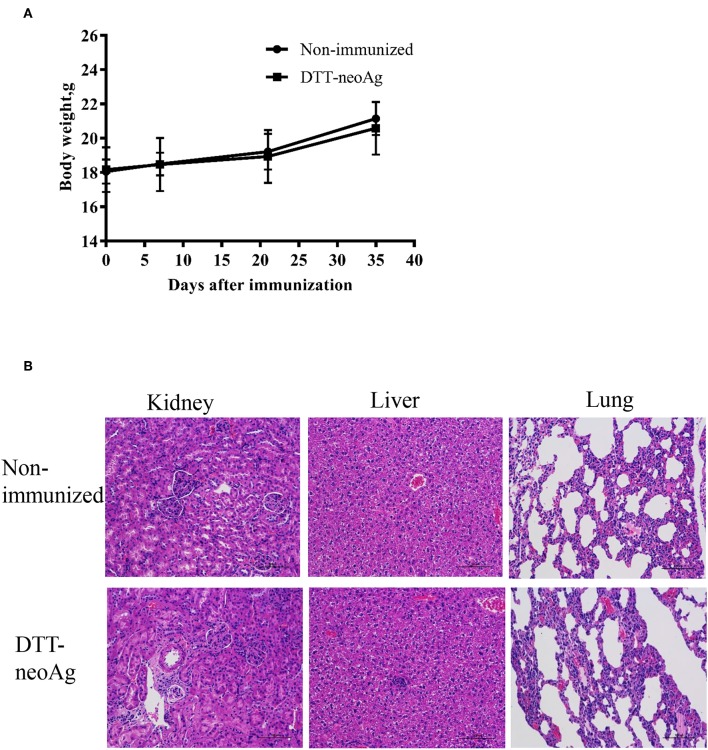
Toxicity evaluation of DTT-neoAg. **(A)** The body weight of the two groups were measured at 7 days after each immunization. **(B)** Morphology evaluation by H&E. Two hundred and ten days after the third immunization, the kidneys, livers, and lungs of DTT-neoAg-immunized mice were collected and analyzed by H&E.

We monitored the appearance and behavior of the immunized mice for 7 months since the beginning of the experiments, and found no significant difference between the DTT-neoAg-immunized and non-immunized mice in terms of weight loss, ruffing of fur, and feeding behavior (Figure 8A). Furthermore, no significant changes were observed in histologies of kidneys, livers, and lungs (Figure 8B) based on H&E staining of the tissues thereof, indicating that the vaccine was safe at least in a short term after treatment. Longer term of safety concern warrants further assessment.

## Discussion

Self-antigens shared by cancer patients have been used in the development of cancer vaccine, yet the clinical outcomes were disappointing (31). One reason is that the self-antigens usually elicit low immune responses as they are subjected to central tolerance (15). Another reason is that they are usually expressed in normal tissues although at very low levels. Therefore, the antigens are not entirely specific to the tumor (15). So far, only one therapeutic vaccine (sipuleucel-T) has been approved by the US Food and Drug Administration for the treatment of prostate cancer (37). Recently, cancer vaccines targeting multiple tumor neoantigens have demonstrated remarkable therapeutic outcomes by virtue of higher specificity (12, 13). However, the majority of tumor neoantigens are weak immunogens (2). To achieve sufficient therapeutic benefits, a large number of neoantigens are required for the vaccine design (12), which limit their application to those cancers having high mutation loads. In this study, we show that fusion of neoantigens to a Th epitope carrier protein, DTT, can enhance the immunogenicity of weak neoantigens as well as antitumor efficacy thereof. The effects of tumor regression conferred by the fusion vaccine were comparable to those of highly immunogenic neoantigen vaccines (7–10). Therefore, our strategy significantly expand the pool of effective vaccine targets, which should benefit cancer patients with intermediate/low mutation burdens such as Ovary cancer, Prostate cancer, low grade Glioma, Chronic lymphocytic leukemia, and Acute myeloid leukemia (2, 38, 39).

CTL responses play pivotal roles in the success of antitumor immunity by vaccination. Current neoantigen-based cancer vaccines confer therapeutic benefits largely through neoantigen-specific CD4^+^ T cells (8). Various strategies have been attempted to improve CTL responses such as nanomaterial-based as well as DNA-based vaccination. Nanomaterial vaccines promote antigen uptake and prolong the presentation time on APC (9, 10, 39), while DNA vaccines promote antigen processing and presentation (11, 40). Nevertheless, these strategies have not been successfully used for the weak neoantigens. DTT possesses several T help epitopes which enhance both antibody and CTL responses (41). It is well-recognized that interactions of the B cells with activated CD4^+^ Th cells is required for induction of high affinity antibody responses (42). The priming, expansion, and memory formation and survival of CD8^+^ cytotoxic T lymphocytes are known to require CD4^+^ T cells which help increase expression level of antigen-presentation machinery, costimulatory molecules and cytokines by antigen-presenting cells (APCs) (43–45). We show here that DTT stimulate neoantigen-specific CTL responses, and confer therapeutic benefits in a mouse melanoma model. Of note, the Th epitopes of DTT are recognized by over 70% of human population (41), which indicates that the fusion strategy would benefit a large population of cancer patients.

Diphtheria toxoid and CRM197, a catalytic inactive mutant of Diphtheria toxin have long been used as a vaccine carrier and adjuvant in clinical applications with excellent safety records. Since DTT is a non-toxic domain of the toxin molecule, its safety would not be of great concern as a vaccine adjuvant. It has been shown that pre-immunization of diphtheria toxoid promotes DC migration and recruitment to lymph nodes thereby improves the anti-tumor effect of vaccines (46). Since many people have previously been immunized with DT, DTT-containing vaccine would rapidly induce a memory recall response of CD4^+^ T cells (47). These could help neoAg rapidly elicit antigen specific CTL responses. Our data demonstrated that DTT-neoAg design is easier and more feasible for clinical cancer immunotherapy than current neoantigen-based vaccine approaches.

## Data Availability Statement

All datasets generated for this study are included in the manuscript/Supplementary Files.

## Ethics Statement

All experiments involving mice were performed according to Shanghai Jiao Tong University Experimental Animal Center guidelines. The experimental protocol involving animals were reviewed and approved by Shanghai Jiao Tong University Laboratory Animal Ethics and Use Committee.

## Author Contributions

YZ, LD, and RL conceived the experiments. YZ, ZL, YW, and HC performed experiments. YZ and LD wrote the manuscript.

### Conflict of Interest

LD and RL were employed by company Shanghai HyCharm Inc., Shanghai, China. YZ and RL have a patent “一种靶向癌症变异肽的免疫制 剂及应用” (Immunological preparations and application targeting cancer mutant peptides) pending.
