# Supplementary material for: The Immunogenicity and Anti-tumor Efficacy of a Rationally Designed Neoantigen Vaccine for B16F10 Mouse Melanoma
Source: Front Immunol. 2019 Nov 5;10:2472. doi: 10.3389/fimmu.2019.02472 (PMC6848027; doi:10.3389/fimmu.2019.02472)
Supplement: Supplementary file 1 [file Data_Sheet_1.docx]

***Supplementary Material***

**Supplementary table 1. Primers used for vector construction**

| Name | Sequence |
| --- | --- |
| DTT-F | CTGGGATCCataaatcttgattgggatgtca |
| DTT-R | ACCACCAGAACCACCACCACCGGGACGATTATACGAATTATG |
| neoAg-F | cataattcgtataatcgtcccGGTGGTGGTGGTTCTGGTGGT |
| neoAg-R | CCGCTCGAGTTACATGTAGTTGTCTTTCAGA |
| CTB-neoAg-F | CCGGTGGAGGCGGGTCCTCCGGATGGCTGCCGCAGGCTAAAGTT |
| CTB-neoAg-R | CTGATCAGTTTTTGTTCGGATCCCATGTAGTTGTCTTTCAGACG |

In DTT-F, *BamH I* site is underlined. In neoAg-R, *Xho I* sit is underlined. In CTB-neoAg-F, *BspE I* site is underlined. In CTB-neoAg-R, *BamH I* site is underlin

**Supplementary table 2. Primers used for Quantitative PCR assay**

| Name | Sequence |
| --- | --- |
| mIFN-γ-F | ACAGCAAGGCGAAAAAGGATG |
| mIFN-γ-R | TGGTGGACCACTCGGATGA |
| mIL-12-F | AGACATCACACGGGACCAAAC |
| mIL-12-R | CCAGGCAACTCTCGTTCTTGT |
| mIL-4-F | GGTCTCAACCCCCAGCTAGT |
| mIL-4-R | GCCGATGATCTCTCTCAAGTGAT |
| mIL-10-F | GCTCTTACTGACTGGCATGAG |
| mIL-10-R | CGCAGCTCTAGGAGCATGTG |

**Supplementary table 3. The mutations of neoantigens**

| Label | Gene | Gene ID | cDNA mutation | DNA sequence | Protein  ID | Amino Acid  mutation | Neoantigen peptide |
| --- | --- | --- | --- | --- | --- | --- | --- |
| W1 | Pi4k2b | 67073  (991-1071) | G1031A | TGGCTTCCTCAAGCAAAGGTTCCTTTTTCTGAAGAGACTC**G**AAACTTGATTCTACCCTATATTTCTGACATGAACTTTGTA | AAH62144.1  (331-357) | R344Q | WLPQAKVPFSEET**R**NLILPYISDMNFV |
| M1 |  |  |  | TGGCTTCCTCAAGCAAAGGTTCCTTTTTCTGAAGAGACTC**A**AAACTTGATTCTACCCTATATTTCTGACATGAACTTTGTA |  |  | WLPQAKVPFSEET**Q**NLILPYISDMNFV |
| W2 | Ddb1 | 13194  (1273-1353) | C1312A | TTGGTGCTCTCTTTTGTGGGCCAGACAAGAGTTCTCATG**C**TAAACGGAGAGGAAGTGGAAGAGACAGAACTGATGGGCTTT | NP_056550.1  (425-451) | L438I | LVLSFVGQTRVLM**L**NGEEVEETELMGF |
| M2 |  |  |  | TTGGTGCTCTCTTTTGTGGGCCAGACAAGAGTTCTCATG**A**TAAACGGAGAGGAAGTGGAAGAGACAGAACTGATGGGCTTT |  |  | LVLSFVGQTRVLM**I**NGEEVEETELMGF |
| W3 | Pcdhga11 | 93723  (205-285) | G244C | AGGGGACAATCGCAGCTTTTCTCTCTGAATCCGCGAGGC**G**GCAGCTTGGTCACTGCAGGTAGGATCGACCGTGAAGAGCTA | NP_291072.1  (69-95) | G82R | RGQSQLFSLNPRG**G**SLVTAGRIDREEL |
| M3 |  |  |  | AGGGGACAATCGCAGCTTTTCTCTCTGAATCCGCGAGGC**C**GCAGCTTGGTCACTGCAGGTAGGATCGACCGTGAAGAGCTA |  |  | RGQSQLFSLNPRG**R**SLVTAGRIDREEL |
| W4 | Atp11a | 50770  (1525-1605) | G1566C | TCCTCGCCTGATGAGGTTGCACTGGTCGAAGGCGTGCAGAG**G**CTTGGATTCACGTACCTGAGGCTGAAGGACAATTACATG | AAI38716.1  (509-535) | R522S | SSPDEVALVEGVQ**R**LGFTYLRLKDNYM |
| M4 |  |  |  | TCCTCGCCTGATGAGGTTGCACTGGTCGAAGGCGTGCAGAG**C**CTTGGATTCACGTACCTGAGGCTGAAGGACAATTACATG |  |  | SSPDEVALVEGVQ**S**LGFTYLRLKDNYM |

**Abbreviations**

| Abbreviation | Description |
| --- | --- |
| DTT | Diphtheria toxin T domain |
| CTB | Cholera toxin B subunit |
| DTT-neoAg | DTT-neoantigen |
| DTT-wtAg | DTT-Wild type |
| CTB-neoAg | CTB-neoantigen |
| CTB-wtAg | CTB-Wild type |
| neoAg-pep | Neoantigen peptide |
| TILs | Tumour infiltrating leucocytes |
| CTLs | Cytotoxic T lymphocytes |
| BMDCs | Bone marrow-derived dendritic cells |
| Alum | Aluminum hydroxide |
| GST | Glutathione S-transferase |
| IPTG | Isopropyl-ß-D-thiogalactoside |
| GM-CSF | Granulocyte-macrophage colony stimulating factor |
| LDH | Lactate dehydrogenase |
| H&E | Hematoxylin and eosin |
| SD | Standard deviation |

**FIGURE S1**


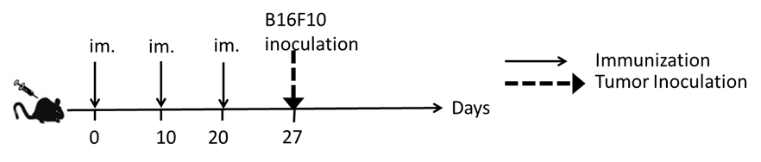


**A**

**C**

**B**


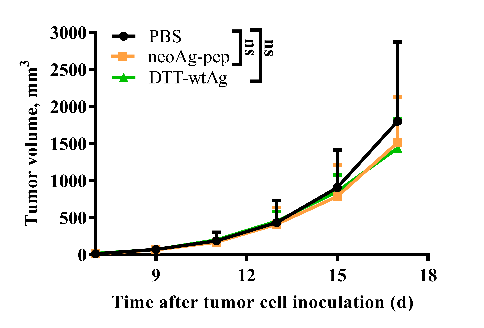

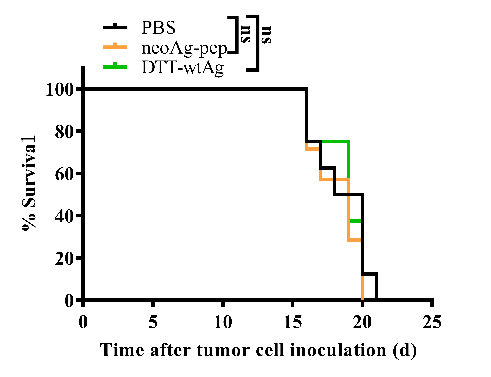


**Figure S1. Anti-tumor effects of neoAg peptide and DTT-wtAg in the prophylactic mouse melanoma model.** **(A)** The treatment schedule. C57BL/6 mice (n = 6-8) were immunized with PBS or neoAg-pep or DTT-wtAg formulated with Alum + CpG on day 0, day 10 and day 20. 7 days after the third immunization, 1×10^5^ B16 F10 cells were s.c. administered into the right flank of the mice. Green line: DTT-wtAg-treated mice, yellow line: neoAg-pep-treated mice, and black line: PBS-treated mice. **(B)** The tumor growth curves. The data are shown in means ± SEM, ns: P > 0.05, Student’s T test. **(C)** The Kaplan-Meier survival plot. The statistical significance was determined by Log-rank test. ns: P > 0.05.

**FIGURE S2**


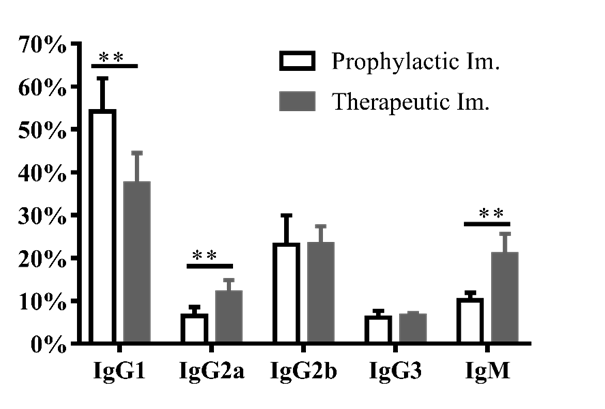


**A**

**Figure S2. Comparison of anti-neoAg antibody subclasses from DTT-neoAg-treated mouse in the prophylactic vaccination and the therapeutic vaccination.** The sera from DTT-treated mice in prophylactic model were collected 7 days after the third immunization. The mouse sera from the therapeutic model were collected from mice challenged with 2.5×10^4^ B16F10 tumor cells 7 days after the third immunization. **(A)** Anti-neoAg antibody subclasses of DTT-neoAg-treated mice were determined by ELISA with the sera diluted at 1:100. Analyses of the percentage of each antibody subclass among total IgGs. **: P < 0.01 by Student’s T test.

**FIGURE S3**


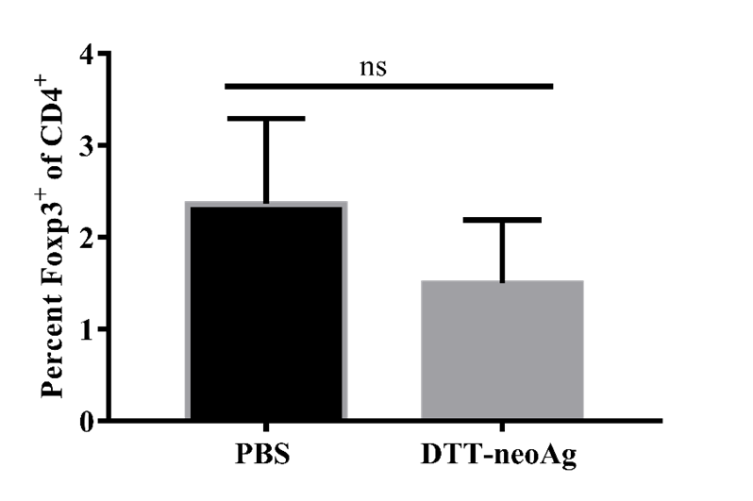


**A**

**Figure S3. Foxp3^+^/CD4^+^ ratio in TILs. (A)** The TILs from DTT-neoAg or PBS vaccinated mice were isolated and stained with anti-CD3ε-APC, anti-CD4- FITC. After permeabilization, intracellular cytokines were stained with anti-Foxp3-PE antibody and analyzed by flow cytometry. The statistical significances were determined by Student’s T test. ns: P > 0.05.
